# Traumatic Brain Injury Leads to Accelerated Atherosclerosis in Apolipoprotein E Deficient Mice

**DOI:** 10.1038/s41598-018-23959-2

**Published:** 2018-04-04

**Authors:** Jintao Wang, Enming Su, Hui Wang, Chiao Guo, Daniel A. Lawrence, Daniel T. Eitzman

**Affiliations:** University of Michigan, Department of Internal Medicine, Cardiovascular Research Center, Ann Arbor, Michigan USA

## Abstract

Traumatic brain injury (TBI) has been associated with atherosclerosis and cardiovascular mortality in humans. However the causal relationship between TBI and vascular disease is unclear. This study investigated the direct role of TBI on vascular disease using a murine model of atherosclerosis. Apolipoprotein E deficient mice were placed on a western diet beginning at 10 weeks of age. Induction of TBI or a sham operation was performed at 14 weeks of age and mice were sacrificed 6 weeks later at 20 weeks of age. MRI revealed evidence of uniform brain injury in all mice subjected to TBI. There were no differences in total cholesterol levels or blood pressure between the groups. Complete blood counts and flow cytometry analysis performed on peripheral blood 6 weeks following TBI revealed a higher percentage of Ly6C-high monocytes in mice subjected to TBI compared to sham-treated mice. Mice with TBI also showed elevated levels of plasma soluble E-selectin and bone marrow tyrosine hydroxylase. Analysis of atherosclerosis at the time of sacrifice revealed increased atherosclerosis with increased Ly6C/G immunostaining in TBI mice compared to sham-treated mice. In conclusion, progression of atherosclerosis is accelerated following TBI. Targeting inflammatory pathways in patients with TBI may reduce subsequent vascular complications.

## Introduction

Traumatic brain injury (TBI) is a common cause of morbidity and mortality worldwide^1^. In the United States, there are an estimated 5.3 million people living with a TBI-related disability^2^. TBI commonly leads to neurocognitive deficits, however, other systemic effects have also been associated with TBI. Cardiovascular effects include stress-related cardiomyopathy^3^, arrhythmias^4^, ECG changes^5^, and increased myocardial reactive oxygen species^6^. In a study of TBI in veterans, TBI was also associated with the severity of coronary artery calcification measured by electron beam computed tomography^7^. Importantly, TBI was independently associated with cardiovascular mortality^7^. These studies indicate there may be a chronic effect of TBI on atherosclerosis. However, whether there exists a direct causal relationship between TBI and systemic atherosclerosis remains unclear. The current preclinical study was performed to determine whether TBI was causally related to progression of atherosclerosis.

## Methods and Materials

### Animals

Male apolipoprotein E–deficient (*ApoE*^−/−^) on the C57BL6/J strain background were purchased from Jackson Laboratory (Bar Harbor, Maine) at 8 weeks of age. Mice were housed under specific pathogen-free conditions in static microisolator cages with tap water ad libitum in a temperature-controlled room with a 12:12-hour light/dark cycle. At 10 weeks of age mice were started on a Western diet (TD88137, Harlan, WI) and at 14 weeks of age, mice were randomly allocated to the TBI or sham procedures. From our previous studies, the mean value of lesion area on aortic trees is estimated at 5% with standard deviation of about 2%^8^. In a study in veterans, mild traumatic brain injury was associated with 2-fold increased CV mortality rate and 4-fold increased in coronary calcification^7^. To estimate the sample size, a difference of 50% was anticipated With a default value for alpha of 0.05 and power of 80% a sample size of 7 for each group was considered sufficient to test hypothesis (http://clincalc.com/stats/samplesize.aspx). Thus, seven mice were randomly assigned to the sham operation group and eight mice were assigned to the TBI group.All animal use protocols complied with the Principle of Laboratory and Animal Care established by the National Society for Medical Research and were approved by the University of Michigan Committee on Use and Care of Animals.

### Model of TBI

To induce TBI, male *ApoE*^−/−^ mice were anesthetized with 2% isoflurane and placed in a stereotactic frame (Kopf,Tujunga,CA, USA) as previously described^9,10^. Briefly, a 5 mm circular craniotomy, centered near the bregma, was made and then a controlled cortical impact (CCI) was delivered to the midline at an impact speed of 3.00 m/s, tissue displacement of 1.1 mm, and impact duration of 50 ms. Following impact, the circular bone fragment from the craniotomy was glued back to the cranial window. The sham procedure was identical except for the delivery of the CCI.

### MRI imaging

24 hours following CCI or sham operation, animals were anesthetized with 2% isoflurane for T2 scanning (7.0 T Varian MR,183 mm horizontal bore, Varian, Palo Alto, CA, USA) as described previously^9^.

### Blood Pressure Measurement

Blood pressure was measured 3 weeks after CCI or sham operation in non-anesthetized, trained mice by tail plethysmography using the BP-2000 Blood Pressure Analysis System (Visitech System, Apex, NC) as previously described^11^.

### Histological Analysis

Quantification of atherosclerosis was performed as previously described^11^. Briefly, mice were euthanized under IP pentobarbital anesthesia (100 mg/kg), and arterial trees were perfused at physiological pressure and fixed in 10% zinc formalin. Arterial trees were then stained with Oil-red-O and pinned on wax trays to quantify the atherosclerotic surface area occupied in the aortic arch, brachiocephalic, common carotid and subclavian arteries. The lesion area was expressed as a percentage of total surface area examined. Paraffin-embedded hearts, which included aortic valves, were also sectioned for lesion analysis. A series of 5 μm sections were obtained at the level of the aortic sinus and 4 cross sections were analyzed from each mouse at each site. Sections were stained with hematoxylin and eosin for quantification of lesion area normalized by adjacent medial area of aorta to control for possible tangential sectioning^11,12^. The lesion area was defined as the area between the endothelial cell layer and internal elastic lamina.

For bone marrow analysis, proliferating cells and Ly6C/G positive cells in paraffin embedded sections were identified with a rabbit anti-PCNA polyclonal antibody (1:50) (Santa Cruz biotechnology, Dallas, TX and a rat anti-mouse Ly6C/G monoclonal antibody (1:20) (BD Biosciences, San Jose, CA), respectively, followed by detection with biotin-conjugated secondary goat anti-rat IgG (1:100) (Accurate Chemical & Scientific Corp., Westbury, NY). For plaque composition analysis, collagen content was examined with Sirius red (Sigma, St. Louis, MO).The macrophage and actin content were quantified with corresponding antibodies to Mac-3 (1:100, BD Biosciences, San Jose, CA), or α-smooth muscle cell actin (1:1000, Cedarlane Laboratories, Burlington, NC). Ly6C/G immunostaining was performed as described above for bone marrow with the rat anti-mouse Ly6C/G monoclonal antibody (1:20) followed by detection with the biotin-conjugated secondary goat anti-rat IgG (1:100). Negative controls consisted of tissues handled identically to experimental samples except that the primary antibody was omitted. The detection system was streptavidin-HRP and endogenous peroxidase was quenched with hydrogen peroxide. Microwave antigen retrieval methods were used. Sections were counterstained with hematoxylin. Positively stained cells were counted manually from three fields in each section using NIH ImageJ software and expressed as number of cells per field area. Tyrosine hydroxylase was identified with a rabbit polyclonal anti-tyrosine hydroxylase antibody (1:200; EMD Millipore, Billerica, MA). Positive staining area was analyzed from three fields in each section and expressed as percentage of the total area. All images were analyzed by automated detection of positive stained area using Nikon MetaMorph software.

### Measurements of Plasma Samples

Plasma samples were collected via terminal heart puncture bleeding 6 weeks after TBI or sham operation. Circulating concentrations of soluble E-selectin (sE-sel) was measured with an ELISA kit following manufacturers’ instructions (R&D Systems, Minneapolis, MN). Cholesterol was measured in the Chemistry Core of the Michigan Diabetes Research and Training Center using Enzymatic-Colorimetric kits (Roche, Indianapolis, IN).

### Cell counts and flow cytometry

Circulating white blood cell counts were measured with a Hemavet 950 haematology system (Drew Scientific, Miami Lakes, FL) from blood samples obtained at 6 weeks of age. Flow cytometry was performed with a Gallios Flow Cytometer (Beckman Coulter, Indianapolis, IN) using antibodies to CD11b, CD115, Ly6C and Ly6G (BD Biosciences, San Jose, CA). Fluorescence minus one controls (FMO’s) were used for each measurement.

### Statistical analysis

All data are presented as mean ± standard deviation. Statistical analysis was carried out using GraphPad Prism. Shapiro-Wilk normality test was used for normal distribution testing. Results were analyzed using 2-tailed t-tests for comparison between two groups.

### Ethics Statement

All procedures complied with the Principles of Laboratory and Animal Care established by the National Society for Medical Research and were approved by the University of Michigan Committee on Use and Care of Animals.

## Results

### Effect of TBI on baseline parameters in *ApoE*^**−**/**−**^ mice

**Figure 1:**
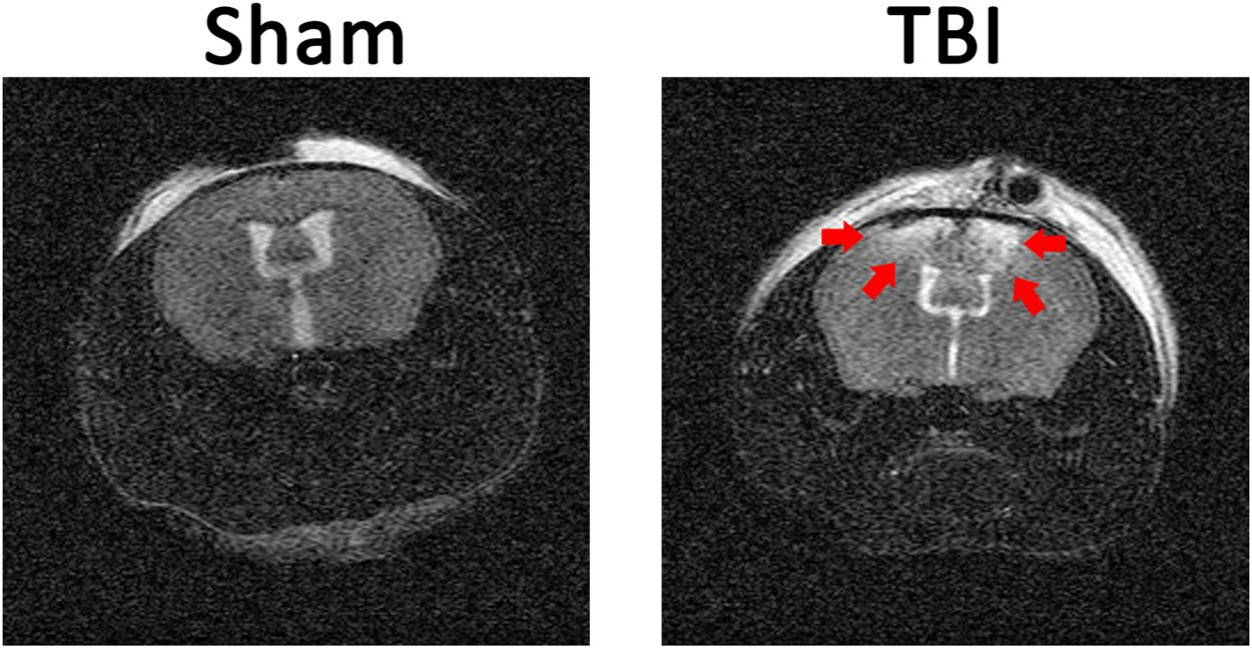
Magnetic resonance imaging (MRI) of brain injury. Apolipoprotein E deficient mice underwent a sham operation or a controlled cortical impact to produce traumatic brain injury (TBI) at 14 weeks of age. MRI was performed 24 hours following injury. Injured area is marked by arrows.

**Figure 2:**
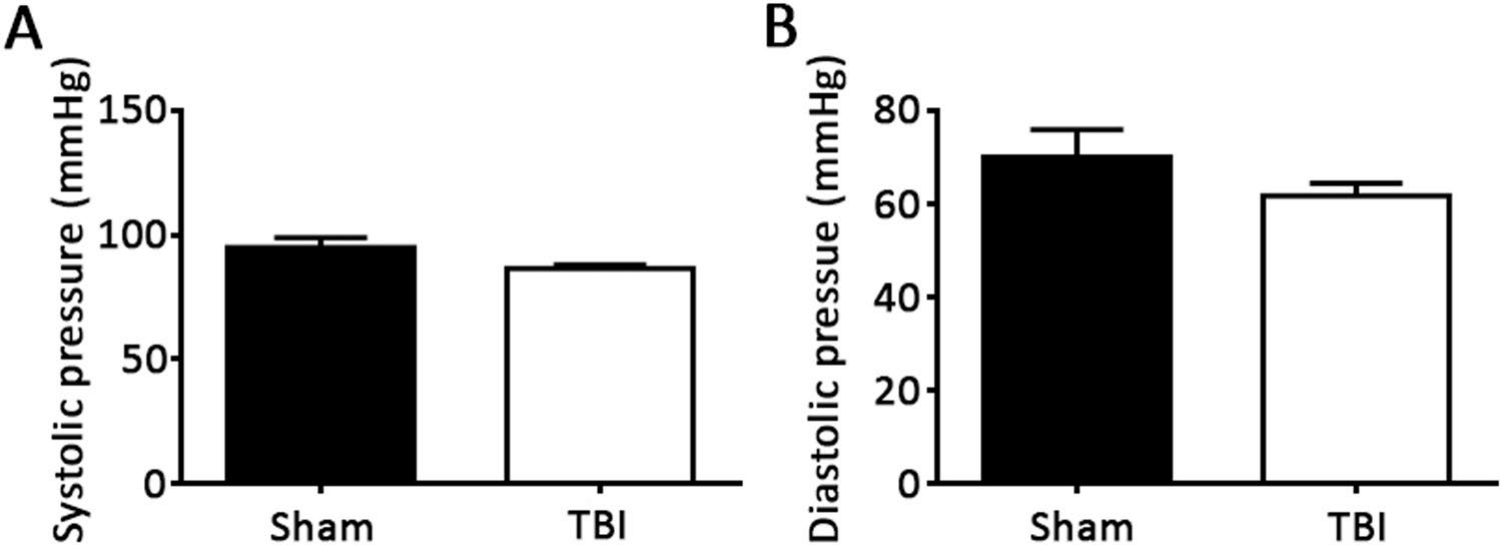
Blood pressure following TBI. Systolic (**A**) and diastolic blood pressures (**B**) were measured in non-anesthetized, trained mice by tail plethysmography 3 weeks after TBI or sham operation. N = 7 for sham group and 8 for TBI group. p = 0.10 (2-tailed t-test) for systolic pressure and 0.25 (2-tailed t-test) for diastolic pressure.

At 10 weeks of age, *ApoE*^−/−^ mice were started on a western diet to induce hyperlipidemia and accelerate the development of atherosclerosis. Four weeks later mice underwent a TBI or sham control procedure. Brain injury was quantified with MRI 24 hours after the surgery. Injured volume was 8.00 ± 1.31% of total brain volume while no injury was detected in a sham group that underwent the same operation without CCI (Fig. 1). Mice recovered quickly from the procedure and demonstrated grossly normal activity and eating behavior. Consistently, body weights were similar between the TBI and sham groups of mice 6 weeks following the procedure (28.59 ± 3.39 grams in sham group vs. 29.34 ± 1.51 grams in TBI group, p = 0.20). There were also no signficant differences in total cholesterol between the 2 groups of mice (497.1 ± 174.0 mg/dL in sham group vs 651.4 ± 116.5 mg/dL in TBI group, p = 0.07). Since brain injuries have been associated with surges of sympathetic activity that could affect blood pressure, tail-cuff plethysmography was used to measure blood pressure 3 weeks following injury in non-anesthetized mice. To ensure reliable and stable blood pressure measurements, mice were first trained for seven consecutive days and all blood pressure measurements were performed in the morning. As shown in Fig. 2, there were no differences in systolic or diastolic blood pressures between the TBI or sham groups of mice.

### Evidence of myeloid activation following TBI

**Figure 3:**
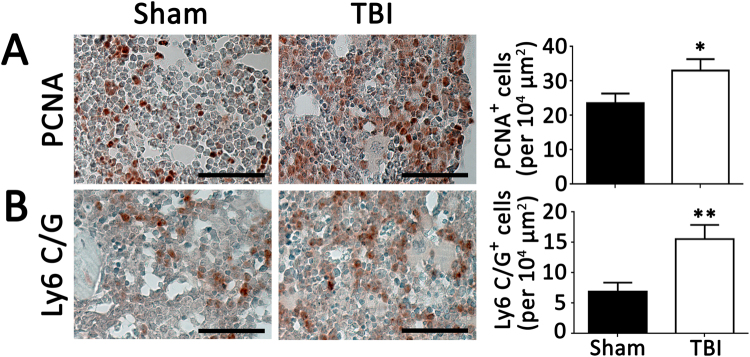
Proliferating and myeloid cellular components of bone marrow. Quantification of proliferating cells (PCNA positive, **A**), and Ly6 C/G positive cells (**B**) 6 weeks following TBI or sham operation. N = 7 for sham group and 8 for TBI group. Scale: 50 µm. *p < 0.05 (2-tailed t-test); **p < 0.01 (2-tailed t-test).

**Figure 4:**
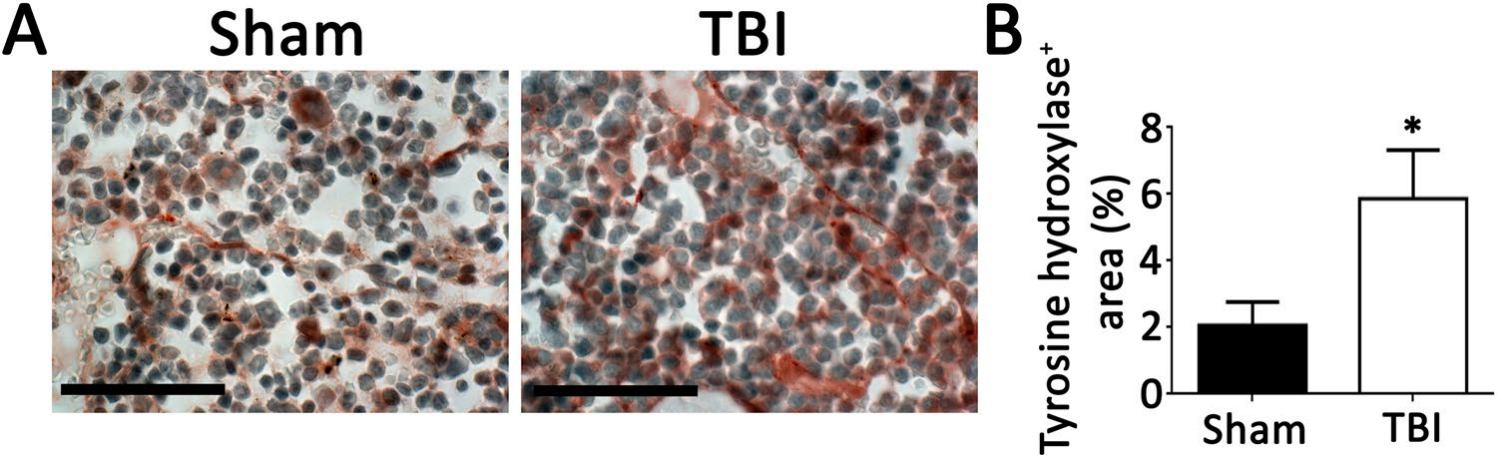
Tyrosine hydroxylase in bone marrow. As a marker of sympathetic activity, immunostaining for tyrosine hydroxylase was performed on bone marrow 6 weeks following TBI or sham operation. N = 7 for sham group and 8 for TBI group. Scale: 50 µm. *p < 0.05 (2-tailed t-test).

**Figure 5:**
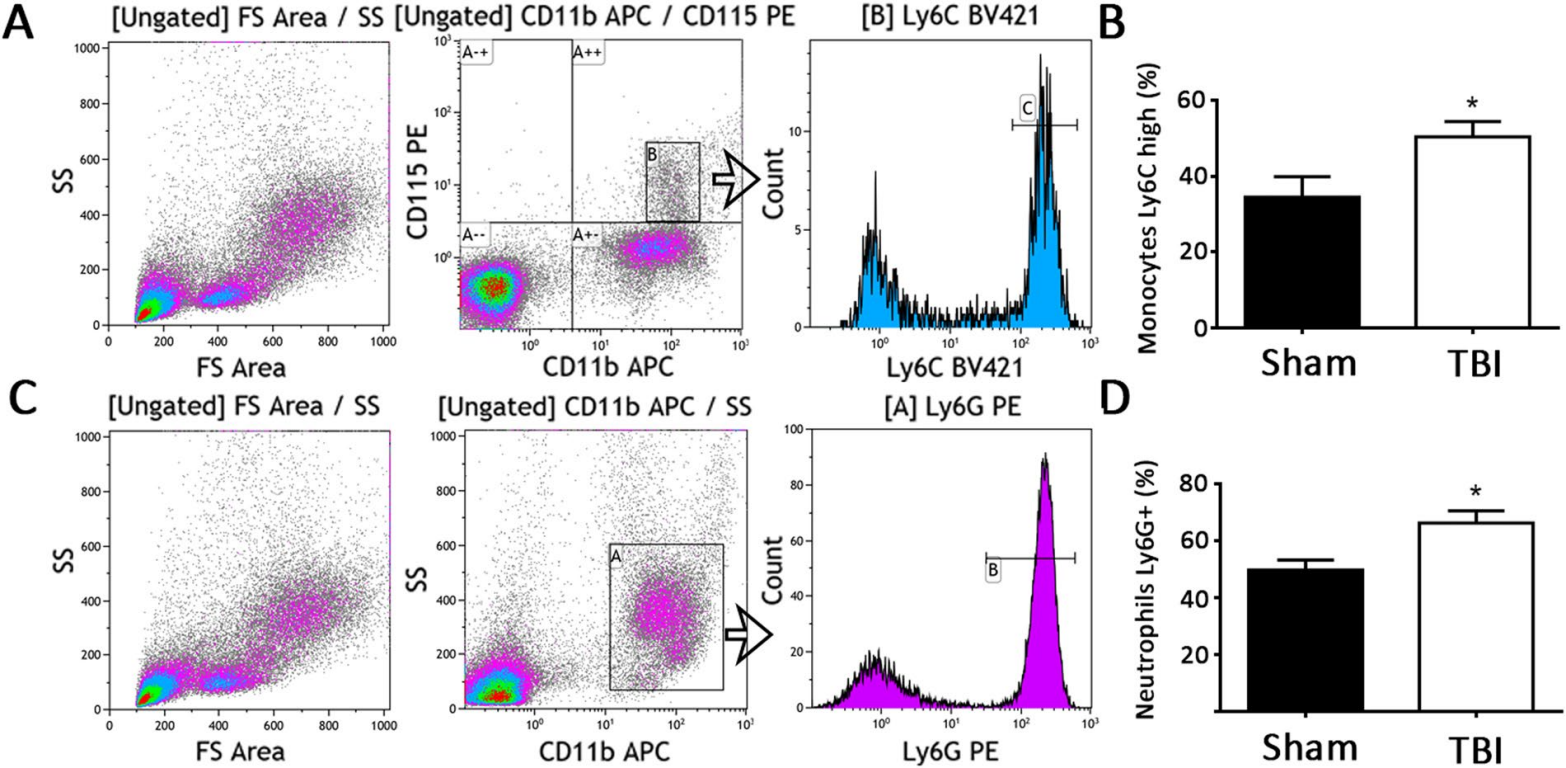
Circulating monocyte and neutrophil phenotype. (**A**) Gating strategy for Ly6C high monocytes. (**B**) Ly6C high monocyte percentage of total monocytes 6 weeks following TBI or sham operation. (**C**) Gating strategy for Ly6G positive neutrophils. (**D**) Ly6G positive neutrophil percentage of total neutrophils 6 weeks following TBI or sham operation. N = 7 for sham group and 8 for TBI group. *p < 0.05 (2-tailed t-test).

**Figure 6:**
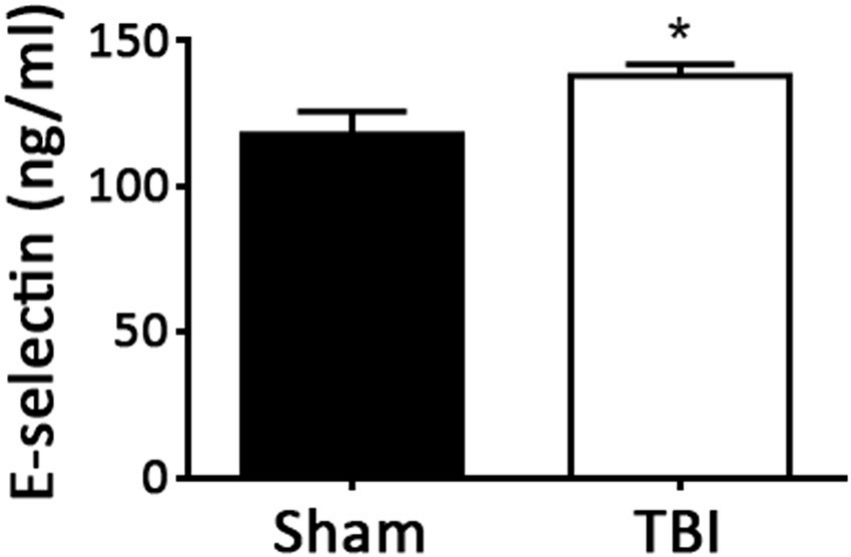
Plasma soluble E-selectin levels. Plasma levels of sE-selectin were measured 6 weeks following TBI or sham operation. N = 7 for sham group and 8 for TBI group. *p < 0.05 (2-tailed t-test).

To investigate potential systemic proinflammatory effects of TBI, the bone marrow was analyzed for evidence of myeloid expansion. Immunostaining of bone marrow cross sections for myeloid markers revealed greater staining for proliferating cells and Ly6C/G positive cells in TBI mice compared to sham mice (Fig. 3). Higher tyrosine hydroxylase staining in bone marrow of mice with TBI compared to mice with sham operation was also evident, indicating sympathetic activation (Fig. 4). Analysis of circulating blood cells revealed similar total white blood cell counts, as well as neutrophils, lymphocytes and monocytes 6 weeks after injury between the 2 groups of mice (Table 1). However, examination of subpopulations of leukocytes by flow cytometry revealed that circulating CD11b^+^, CD115^+^, Ly6C^high^ monocyte and CD11b^+^, Ly6G^+^ neutrophil percentages were higher in the TBI group compared to the sham group (Fig. 5). Consistent with increased systemic endothelial adhesiveness, soluble E-selectin levels were higher in TBI mice compared to sham-operated mice 6 weeks following the TBI (Fig. 6).

**Table 1:** Complete blood counts.

	WBC (K/µl)	NE (K/µl)	LY (K/µl)	MO (K/µl)
Sham	9.88 ± 4.06	4.91 ± 3.98	4.32 ± 0.35	0.34 ± 0.03
TBI	9.71 ± 2.41	5.02 ± 1.89	4.06 ± 1.21	0.32 ± 0.13

### Effect of TBI on atherosclerosis in *ApoE*^**−**/**−**^ mice

**Figure 7:**
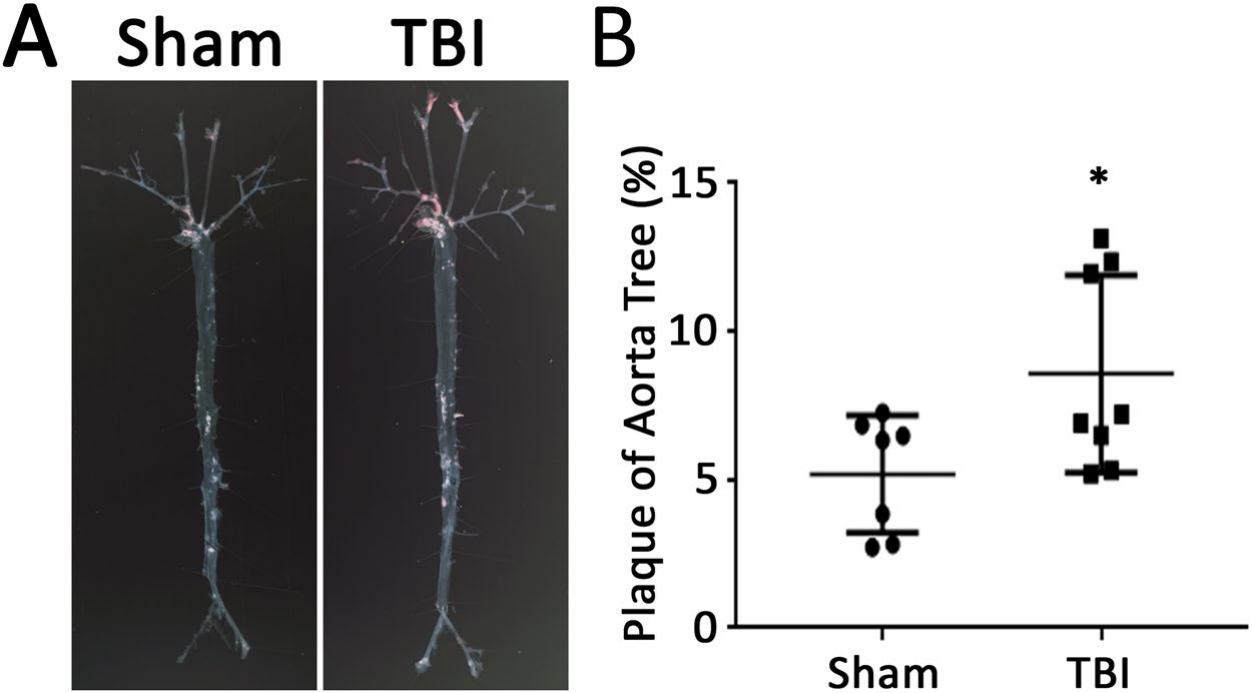
Surface area atherosclerosis involving aorta and major branches. (**A**) Representative photographs of aortic trees 6 weeks following TBI or sham operation. (**B**) Quantification of oil-red-O staining plaque area of aortic trees 6 weeks following TBI or sham operation. N = 7 for sham group and 8 for TBI group. *p < 0.05 (2-tailed t-test).

**Figure 8:**
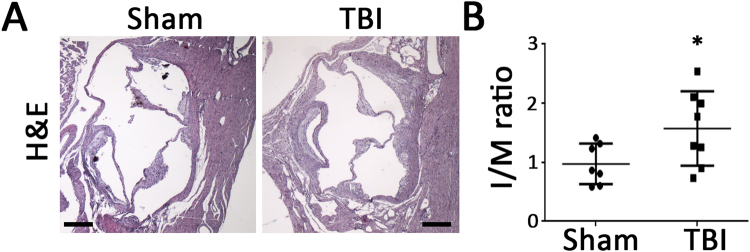
Aortic root lesion cross-sectional area. Representative photomicrographs (**A**) and quantification of lesion area normalized by respective medial area of aorta at aortic root stained with hematoxylin and eosin (**B**) 6 weeks following TBI or sham operation. Scale: 200 µm. N = 7 for sham group and 8 for TBI group. *p < 0.05 (2-tailed t-test).

**Figure 9:**
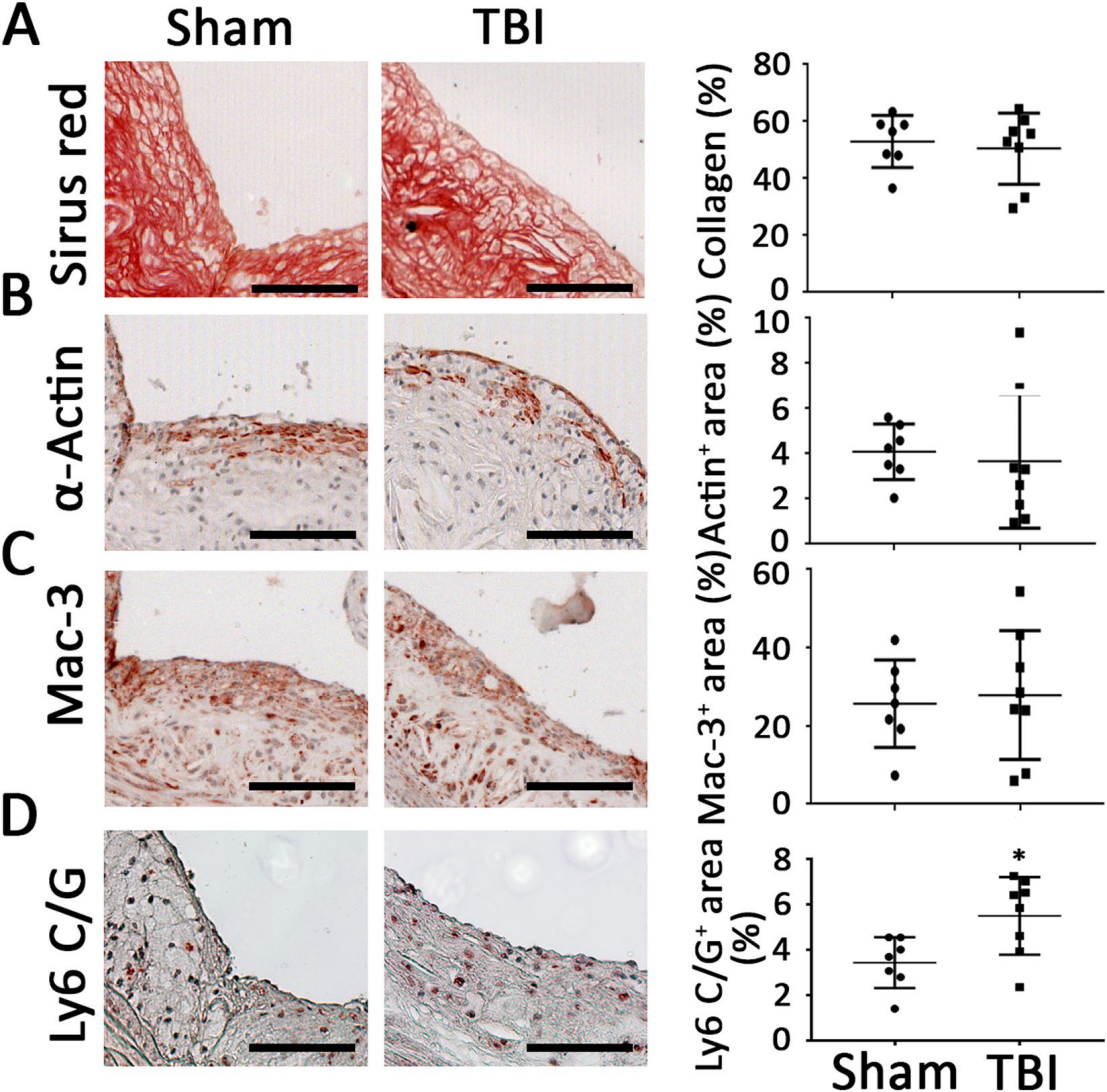
Cellular composition analysis of plaque involving aortic root. Representative photomicrographs and quantification of collagen (**A**, p = 0.66 (2-tailed t-test)), smooth muscle cells (**B**, p = 0.73 (2-tailed t-test)),macrophages (p = 0.76 (2-tailed t-test)) and Ly6 C/G positive staining (**D**, *p < 0.05 (2-tailed t-test)) in aortic root plaque. N = 7 for sham group and 8 for TBI group. Scale: 100 µm.

To determine whether the apparent systemic proinflammatory changes described above following TBI were associated with increased vascular disease, atherosclerosis was quantitated following the TBI or sham procedures. Quantitation of atherosclerosis throughout the aorta and major vessels revealed greater atherosclerosis burden in mice with TBI compared to the sham group as determined by Oil-red-O staining (Fig. 7). Cross-sectional analysis of atherosclerotic plaque area at the level of the aortic valve also revealed increased plaque area in mice with TBI compared to the sham control group (Fig. 8). Immunostaining for smooth muscle cells, macrophages and collagen revealed no difference between the two groups (Fig. 9A–C). However, Ly6 C/G positive staining area was increased in plaques from TBI compared to sham mice (Fig. 9D).

## Discussion

Cardiovascular complications following brain injury are not uncommon and include stress-related cardiomyopathy, arrhythmias, ECG repolarization changes, and increased cardiac reactive oxygen species^13^. These effects may be mediated by catecholamine surges as chronic and paroxysmal sympathetic hyperactivity are common after traumatic brain injury^14^ and sympathetic overactivity is associated with chronic deleterious vascular effects^15^. Consistently, TBI has previously been shown to induce a coagulopathy and endotheliopathy as evidenced by biomarkers and these effects were associated with sympathoadrenal activation^16^ and reflected a poor prognosis. More subtle, chronic sympathetic activity has been demonstrated to play an important role in atherosclerosis. For example, patients suffering myocardial infarction or stroke are at increased risk of a subsequent event within the first year following the incident event^17,18^ and this is associated with elevated inflammatory biomarkers^19,20^. In preclinical models, myocardial infarction and stroke lead to acceleration of atherosclerosis that is associated with enhanced sympathetic nervous system activity and evidence of leukocyte activation^21,22^. It is unknown whether these systemic effects are unique to ischemic injuries following vascular occlusion or whether the responses to injury are organ-specific. Of particular interest, the brain may be an important regulator of subsequent heightened inflammation via bone marrow signaling pathways leading to activation and release of myeloid progenitor cells^23–25^. It is therefore plausible that TBI may also promote a state of chronic, low grade inflammation that could promote atherosclerotic vascular complications. Consistent with this idea, a clinical study of TBI in United States veterans, with a median of 4 year follow up, demonstrated that TBI was strongly associated with the severity of coronary artery calcification as measured by electron beam computed tomography^7^ suggesting that TBI may promote processes involved in atherogenesis. Importantly, there was a marked independent association of TBI with cardiovascular mortality with a relative risk of 2.89 compared to a non-TBI control group, even after adjusting for typical cardiovascular risk factors. These observations indicate there may be a chronic and relatively potent effect of TBI on atherosclerosis. However, whether these findings represent a direct link between TBI and systemic vascular disease or represent other confounding factors that may be present in the TBI group is unclear. Identification of the causal role of TBI on atherogenesis and the potential pathways involved is critical considering the implications for therapeutic intervention in TBI patients. A preclinical model is necessary to address these fundamental questions.

The model of TBI used in this study has been widely utilized to study mechanisms involved in brain injury and repair^26^. Similar to TBI in humans, post-injury cascades are activated that may have systemic chronic effects including apoptosis^27^, inflammation^28^ and oxidative stress^29,30^. Because of the very low mortality rate associated with the CCI model, it has also been used in long term survival studies. Of particular interest, pathophysiological changes continue to occur even 1 year after CCI, including progressive neurodegeneration associated with microglial activation^31^, and compensatory neurologic responses^32^. The model is thus appropriate to study potential effects of TBI on atherosclerosis.

Apolipoprotein E deficient mice are widely utilized to study factors involved in atherosclerosis^33^. Atherosclerosis is greatly accelerated following challenge with a western diet^8,11,34^, enhancing feasibility of atherosclerosis studies and allowing the current study to focus on a timepoint 6 weeks following the TBI.

Differences in cholesterol levels or blood pressures could affect atherosclerosis in this model, however, no differences in either parameter were observed between mice subjected to TBI versus the sham operation. However, evidence of increased sympathetic activity to bone marrow was present as indicated by immunostaining for tyrosine hydroxylase, which has previously been shown to be a sensitive marker for sympathetic innervation^11^.

Bone marrow sympathetic innervation has been implicated in a chronic myeloid pro-inflammatory response following certain injuries^22^. While increases in total leukocyte counts, monocytes, neutrophils, or lymphocytes were not observed in this study 6 weeks following TBI, flow cytometric analysis revealed evidence of monocyte and neutrophil activation. This could be due to sympathetic effects on bone marrow myeloid maturation and/or release, however changes could also be initiated in the spleen or circulation. The mediator(s) responsible for these effects also needs to be investigated further.

Effects of TBI on leukocyte activation could represent a mediator of the increased atherosclerosis observed as it has been previously shown that Ly6C^high^ monocytes are preferentially recruited to sites of atherosclerotic plaque formation^35^ due to increased leukocyte-endothelial adhesive interactions which appears to be the case in this study. Soluble E-selectin levels were measured as an endothelial-specific biomarker of leukocyte-endothelial adhesive interactions^36^. Consistent with increased systemic endothelial adhesiveness, soluble E-selectin levels were higher in TBI mice compared to sham-operated control mice 6 weeks following the TBI. E-selectin has been shown to play a critical role in the acceleration of atherosclerosis following MI^37^.

Regardless of the mechanism(s) involved, this study provides evidence for causal relationship between TBI and subsequent vascular complications. This could be important as TBI could serve as another factor in determining eligibility for statin therapy. Whether statins would be effective in reducing this increased risk would need to be investigated. Ideally, specific mediators of the increased risk associated with TBI could be identified and specifically targeted. This study highlights potential targets related to myeloid activation and leukocyte adhesion. Of particular relevance, recent targeting of inflammatory pathways related to IL-1B signaling has been shown to reduce vascular endpoints in high risk patients^38^. Identification of specific patient populations with activation of inflammatory pathways that would most likely benefit from this therapeutic approach would enhance its feasibility.

In conclusion, TBI promotes atherosclerosis and supports recent clinical studies showing increased cardiovascular risk in TBI patients. Targeting inflammatory pathways in addition to conventional risk factors for atherosclerosis may reduce vascular comorbidities associated with TBI.
